# Saturated fat and human health: a protocol for a methodologically innovative systematic review and meta-analysis to inform public health nutrition guidelines

**DOI:** 10.1186/s13643-023-02209-1

**Published:** 2023-03-14

**Authors:** Bradley C. Johnston, Dena Zeraatkar, Jeremy Steen, Diego Rada Fernandez de Jauregui, Hongfei Zhu, Mingyao Sun, Matthew Cooper, Malgorzata Maraj, Anna Prokop-Dorner, Boris Castro Reyes, Claudia Valli, Dawid Storman, Giorgio Karam, Joanna Zajac, Long Ge, Mateusz J. Swierz, Nirjhar Ghosh, Robin W. M. Vernooij, Yaping Chang, Yunli Zhao, Lehana Thabane, Gordon H. Guyatt, Pablo Alonso-Coello, Lee Hooper, Malgorzata M. Bala

**Affiliations:** 1 Department of Nutrition, College of Agriculture and Life Science, Texas A&M University, College Station, TX USA; 2 Department of Epidemiology and Biostatistics, School of Public Health, College Station, TX USA; 3 Department of Biomedical Informatics, Harvard Medical School, Boston, MA USA; 4 Department of Health Research Methods, Evidence and Impact, McMaster University, Hamilton, ON Canada; 5 Faculty of Health Sciences, McMaster University, Hamilton, ON Canada; 6 Preventive Medicine and Public Health Department, School of Pharmacy, University of the Basque Country/Euskal Herriko Unibertsitatea (UPV/EHU), Vitoria-Gasteiz, Spain; 7 Department of Nutrition, Texas A&M University, College Station, TX USA; 8 Evidence Based Social Science Research Centre, School of Public Health, Lanzhou University, Lanzhou, China; 9 Department of Social Medicine and Health Management, School of Public Health, Lanzhou University, Lanzhou, China; 10 Evidence Based Nursing Centre, School of Nursing, Lanzhou University, Lanzhou, China; 11 Department of Medicine, Faculty of Medicine and Dentistry, University of Alberta, Edmonton, AB Canada; 12 Department of Hygiene and Dietetics, Chair of Epidemiology and Preventive Medicine, Jagiellonian University Medical College, Jagiellonian University Medical College, Krakow, Poland; 13 Department of Medical Sociology, Chair of Epidemiology and Preventive Medicine, Jagiellonian University Medical College, Krakow, Poland; 14 Iberoamerican Cochrane Centre, Biomedical Research Institute San Pau (IIB Sant Pau), Barcelona, Spain; 15 Department of Paediatrics, Obstetrics, Gynaecology and Preventive Medicine, Universidad Autónoma de Barcelona, Barcelona, Spain; 16 Department of Hygiene and Dietetics, Chair of Epidemiology and Preventive Medicine, Faculty of Medicine, Jagiellonian University Medical College, Kraków, Poland; 17 Systematic Reviews Unit, Jagiellonian University Medical College, Krakow, Poland; 18 Department of Adult Psychiatry, University Hospital, Krakow, Poland; 19 Max Rady College of Medicine, University of Manitoba, Winnipeg, MB Canada; 20 Department of Nephrology and Hypertension, University Medical Center Utrecht, Utrecht, The Netherlands; 21 Julius Center for Health Sciences and Primary Care, University Medical Center Utrecht, Utrecht, The Netherlands; 22 The Center of Gerontology and Geriatrics (National Clinical Research Center for Geriatrics), West China Hospital, Sichuan University, Chengdu, China; 23 Department of Medicine, Faculty of Health Sciences, Hamilton, ON Canada; 24 CIBER de Epidemiología Y Salud Pública (CIBERESP), Madrid, Spain; 25 Norwich Medical School, University of East Anglia, Norwich Research Park, Norwich, NR4 7TJ UK

## Abstract

**Background:**

The health effects of dietary fats are a controversial issue on which experts and authoritative organizations have often disagreed. Care providers, guideline developers, policy-makers, and researchers use systematic reviews to advise patients and members of the public on optimal dietary habits, and to formulate public health recommendations and policies. Existing reviews, however, have serious limitations that impede optimal dietary fat recommendations, such as a lack of focus on outcomes important to people, substantial risk of bias (RoB) issues, ignoring absolute estimates of effects together with comprehensive assessments of the certainty of the estimates for all outcomes.

**Objective:**

We therefore propose a methodologically innovative systematic review using direct and indirect evidence on diet and food-based fats (i.e., reduction or replacement of saturated fat with monounsaturated or polyunsaturated fat, or carbohydrates or protein) and the risk of important health outcomes.

**Methods:**

We will collaborate with an experienced research librarian to search MEDLINE, EMBASE, CINAHL, and the Cochrane Database of Systematic Reviews (CDSR) for randomized clinical trials (RCTs) addressing saturated fat and our health outcomes of interest. In duplicate, we will screen, extract results from primary studies, assess their RoB, conduct de novo meta-analyses and/or network meta-analysis, assess the impact of missing outcome data on meta-analyses, present absolute effect estimates, and assess the certainty of evidence for each outcome using the GRADE contextualized approach. Our work will inform recommendations on saturated fat based on international standards for reporting systematic reviews and guidelines.

**Conclusion:**

Our systematic review and meta-analysis will provide the most comprehensive and rigorous summary of the evidence addressing the relationship between saturated fat modification for people-important health outcomes. The evidence from this review will be used to inform public health nutrition guidelines.

**Trial registration:**

PROSPERO Registration: CRD42023387377.

## Background

Non-communicable diseases, including cardiovascular disease (CVD), cancer, and diabetes are responsible for 4 of 5 deaths worldwide [1]. Modifying dietary habits may reduce the incidence of non-communicable diseases, though what constitutes an optimal fat intake and dietary pattern is highly debated.

The health effects of dietary fats are a controversial issue on which experts and authoritative organizations have often disagreed [2, 3]. While some guidelines, for example, have recommended restricting dietary fats to less than 30–35% of total energy intake, others have concluded that reduction of total dietary fats has little effect on improving health outcomes [4–7]. The relationship between saturated fats and cardiovascular disease is another case in point, about which authoritative organizations and experts continue to disagree [8, 9].

Care providers, guideline developers, policy-makers, and researchers use systematic reviews to advise patients on optimal dietary habits, formulate dietary recommendations and policies, and to plan future research [10–12]. While a plethora of systematic reviews on the health effects of dietary fats have been published to date [13], existing reviews have serious limitations. Most systematic reviews, for example, have addressed only one or a few health-related outcomes, whereas dietary recommendations and related policy implementation require consideration of all people-important outcomes (e.g., all-cause, cardiovascular, and cancer mortality, non-fatal stroke and myocardial infarction, cancer incidence, type 2 diabetes, dementia, and quality of life), as well as patient or public health-related values and preferences that bear on dietary recommendations [11, 12, 14]. Further, existing reviews often contain substantial deficiencies. For example, reviews often fail to present absolute effect estimates, which can lead to misinterpretation of findings [15–20]. Equally important, only a handful of reviews have formally and comprehensively evaluated the certainty of evidence for each outcome—a critical step in contextualizing review findings and generating dietary recommendations [15, 19–21], and aside from one dietary guideline on red and processed meat [22], none have used a contextualized approach, as recently recommended by the Grading of Recommendations, Assessment, Development and Evaluations (GRADE) working group [23–25]. Finally, the extent to which risk of bias (RoB) associated with missing participant outcome data reduces the certainty in results [26, 27] also represents a key issue in contextualizing review findings and the generation of dietary recommendations [15]. For instance, evidence suggests that almost one in every three trials with statistically significant results lose significance when making plausible assumptions about the outcomes of participants with missing data [26].

We propose a methodologically innovative systematic review to summarize existing evidence on reducing or replacing dietary saturated fat with other nutrients (mono- or polyunsaturated fat, carbohydrates, protein) and important health outcomes. This work will improve upon the limitations of previous reviews and guidelines by addressing a comprehensive list of people-important single outcomes that patients and members of the public can better understand (rather than composite outcomes), analyze RoB using a nutrition specific assessment [14, 28] as well as tools for assessing missing data [27, 29], and calculating absolute effect estimates and evaluating the certainty of these estimates using the GRADE fully contextualized approach [15, 24].

## Methods

### Research question

Among adult patients and members of the public with or without cardiometabolic conditions, what is the impact of modifying dietary fat intake (lowering saturated fat intake, or lowering saturated fat while increasing polyunsaturated fat and/or increasing monounsaturated fat and/or protein and/or complex carbohydrates) on the risk of critically important health outcomes (e.g., mortality, stroke, myocardial infarction, quality of life)?

### Scope

Our systematic review will summarize foods and diets addressing reduced saturated fats, and the replacement or modification of saturated fats with monounsaturated, polyunsaturated fats (e.g., omega-3 and omega-6), carbohydrates or protein and the relationship with all-cause mortality; cardiovascular mortality; cancer mortality; cardiovascular disease including coronary heart disease and myocardial infarctions; stroke; total cancer incidence; type 2 diabetes; dementia including Alzheimer’s disease; satisfaction with diet; and quality of life in patients with or without cardiometabolic conditions (e.g., previous history of cardiovascular events such as myocardial infarction or stroke, diabetes, hypertension) but without other chronic (e.g., cancer) or infectious conditions.

### Inclusion criteria

We will include randomized controlled trials (RCTs) of individuals or groups (six or more clusters). Randomized trials have to state an intention to reduce saturated fat (SFA) intake via appropriate food or nutrient-based aims, or trial reports have to provide a dietary aim in general, such as reducing total fat or improving heart health (reduced saturated fats and encouraged fruit and vegetables), while also achieving a statistically significant (*p* < 0.05) reduction in saturated fat reduction between the the intervention arm and control arm during the trial period. Eligible interventions have to be low fat dietary advice, supplementation with naturally occurring oils or fats (e.g., food based olive oil or fish), or provision of modified or low-fat foods, as compared to an intake of a higher saturated fat diet, placebo or a control diet higher in fat (e.g., usual diet). Our intended time-point of interest for the duration of the diet intervention will be 2 years (24 months) or more.

We will exclude trials that are formula based (e.g., weight-loss formulas such a NutriSystem), have a pharmacological intervention for weight-loss (e.g., Olestra), or the primary aim to assess weight-loss (experimental arm is calorie restricted while the control arm is ad libitum). If trials employ active interventions such cardiometabolic or smoking cessation medications (e.g., statins, Metformin, Chantix), the study will be eligible if both groups are provided drugs, active intervention. If a trial demonstrated a statistically significant between group reduction in SFA and encouraged active interventions such as physical exercise, or cardiovascular medications in one arm (intervention) with no exercise or medication in the alternative arm (control), we will exclude. We will also exclude observational studies given that over 50,000 patients have been randomized to SFA reduction and replacement interventions, and the available trials have captured all of our people-important outcomes. For the purpose of informing SFA dietary guidelines, we will use the most recent, high-quality systematic reviews of cohort studies (e.g., [30, 31]).

### Search strategy

We will collaborate with an experienced research librarian to search MEDLINE, EMBASE, CINAHL, and registers for reviews including PROSPERO and the Cochrane Database of Systematic Reviews (CDSR) for systematic reviews of RCTs. For reviews, we will run an updated search from the date of the last comprehensive systematic review (e.g., [32]) for new primary studies. Since questions addressed by older reviews are likely to have been also addressed in more recent reviews, we will restrict the search to reviews published from 2015 onward. We will also search the reference lists of included reviews, related publications on PubMed and Google Scholar, clinicaltrials.gov and contact content and research experts in this area to further augment our search. We will also search included studies from previous systematic reviews to ensure the subsequent studies have not been reported with longer follow-up data.

### Screening and study selection

Pairs of reviewers will complete calibration exercises, after which they will perform screening of search results independently and in duplicate. Reviewers will resolve discrepancies by discussion or by adjudication by an expert research methodologist. To start, we will search for systematic reviews that include one or more RCTs that address the association between dietary fats and important health outcomes (above) in adults with or without cardiometabolic conditions, but without other chronic or infectious conditions. In cases in which two or more systematic reviews reporting on the same exposure and outcome have overlapping searches within three months of one another, we will select the systematic review that is the most comprehensive (i.e., includes the most eligible studies). To identify RCTs published after the end of the search used in the most comprehensive systematic review identified, we will run an updated search of MEDLINE, EMBASE, CINAHL, and CDSR from the date of the last comprehensive systematic review.

### Data extraction

Following calibration exercises, reviewers, working independently, in duplicate and using a standardized, piloted tested data extraction form, will extract data from included reviews and primary studies. Reviewers will resolve discrepancies by discussion or by adjudication by an expert research methodologist.

From each original study, we will extract information on the study characteristics (population, intervention, comparator, outcomes) and results. For event (dichotomous) data we will extract numbers of events in each study arm at the last reported time point available during the intervention period, and the number of participants included in each arm. For continuous data, we will extract means, standard deviations (or other variance data), and numbers in each arm at the last reported time point during the intervention period. We will also extract planned dietary composition in both study arms, assessed dietary composition in both arms, longest duration of the intervention, whether the intervention consists of dietary advice, advice plus some food provision, or provision of most foods, cointerventions (pharmaceutical and non-pharmaceutical), data on compliance in both arms, and surrogate outcomes including total serum cholesterol and low density lipoprotein (LDL) cholesterol in both arms at the latest available date during the intervention (during the randomized period) [32].

### Risk of bias

Following calibration exercises, reviewers, working independently and in duplicate, will assess the RoB of eligible primary studies. Reviewers will resolve discrepancies by discussion or by adjudication by an expert research methodologist.

Systematic reviews often have important limitations related to their assessment of RoB, such as the application of tools that do not address all important sources of bias, tools that include constructs unrelated to RoB (e.g., generalizability), or apply RoB tools inconsistently [33]. Hence, in duplicate we will assess the RoB of primary studies de novo using a modification of the Cochrane risk of bias 1.0 instrument for RCTs [34, 35]. Based on our previous use of the tool, it has been modified in a standardized way in order to make the assessment more manageable and specific to nutrition studies [28]. For instance, our modified Cochrane RoB instrument for RCTs uses more comprehensive instructions for all RoB items with clear definitions and examples for low and high RoB, and uses four response options [36]. In particular, to evaluate sequence generation, allocation concealment and blinding, we will first assess if these items were adequately reported (i.e., clearly reported, mostly reported, mostly not reported, clearly not reported) and second, we will evaluate how serious the RoB is using an index of suspicion (i.e., 1 = definitely low risk, 2 = probably low risk, 3 = probably high risk, 4 = definitely high risk). For example, a study may report “double-blinded” without details as to which of the five possible study team members of an RCT where specifically blinded. For both data analysts and data adjudicators we would answer “clearly not reported” and “probably high risk of bias”. We will also modify the selective outcome reporting item to avoid confounding “outcome reporting bias” with “publication bias”, and use the more comprehensive and specific item for selective reporting used in the Cochrane RoB 2.0 tool [37]. Our team has previously successfully used similar modified instruments for the assessment of RoB for nutrition RCTs [28, 38].

### Risk of bias related to missing participant outcome data

Following calibration exercises, reviewers, working independently and in duplicate and using a standardized and piloted tested data extraction form will extract data on missing participant outcome data (MPOD) from included RCTs that address the association between dietary fats and all people-important outcomes. Reviewers will resolve discrepancies by discussion or by adjudication by an expert research methodologist. As needed, we will contact the trialists to ask for available but unreported MPOD in the primary study report.

We will define participant outcome data as ‘‘missing’’ if they are unavailable to the reviewers; that is, unavailable to investigators of the primary studies, or available to the primary study investigators but not included in published reports and not provided after inquiry.

For our primary analysis, we will use a complete-case analysis (sometimes referred to as an available-case analysis) where participants with missing participant outcome data are excluded from both the numerator and denominator when calculating relative and absolute risks. We will subsequently compare the complete-case analysis to a series of sensitivity analysis to explore the impact of missing data on our outcomes and assess the robustness of the effect estimates as suggested using GRADE 17 guidance [26, 27, 29, 39, 40]. To do so, we will assume that the event rate for those participants in the control group who had missing data was the same as the event rate for those participants in the control group who were successfully followed. For the intervention group, we will calculate effects using the following assumed ratios of event rates in those with missing data in comparison to those successfully followed: 1.5:1, 2:1, 3:1, and 5:1 [26].

### Data synthesis and analysis

For our review of RCTs, for each dietary fat and health outcome of interest, based on guidance from the Cochrane Handbook we will conduct de novo meta-analyses comparing lower versus higher intake of saturated fats, and saturated fats replaced or modified with polyunsaturated fatty acids (PUFA), monounsaturated fatty acids (MUFA), protein and complex carbohydrates. Replacement trials will be those wherein participants are asked to reduce their fat or SFA, and authors report evidence of significant decrease in SFA with a corresponding increase in other nutrients (e.g., SFA is reduced by ~ 6% of daily calories [energy], while there is an increase in energy from PUFA (e.g., 4%) and/or MUFA (e.g., 2%). Replacement trials, while more robust if they provide known quantities of specific food interventions to participants, may or may not provide the intervention (e.g., nuts, olive oil). Depending the types of trials and intervention arms (reduction; replacement; modification of fat and macronutrients), we may conduct both standard pairwise comparisons and a network meta-analysis.

For each outcome reported in each review, we will present the intervention, comparator, number of studies and participants, the baseline risk, the absolute and relative effects and the corresponding certainty of evidence. We will use data from GLOBOCAN [41] and the Emerging Risk Factors Collaboration [42] to estimate the baseline and absolute risks for major cardiometabolic and cancer outcomes, respectively. Absolute risks for cardiometabolic outcomes will be estimated over 10.8 years, while cancer outcomes will be estimated over a lifetime [41, 42]. Using these baseline risks, we will calculate the absolute risk reductions for our respective outcomes [43]. Since our review will inform a dietary guideline, wherein decision-makers need to consider evidence from all people-important outcome data, we will use a fully contextualized approach and we will categorize the magnitude of effects as trivial, small but important, moderate or large using guidance from GRADE [24, 25] and the Cochrane Collaboration [44]. Using thresholds for the magnitude of importance developed in consultation with an international dietary guideline panel on red and processed meat [22], we will use the following categorization. For fatal outcomes, ≤ 10 events per 1000 will be considered a trivial (unimportant) effect size, 11–25 per 1000 will be considered a small but important effect, and 26–40 per 1000 will be considered moderate. For non-fatal outcomes, ≤ 20 per 1000 will be considered trivial, 21–40 per 1000 will be considered small but important, and 41–60 per 1000 will be considered moderate. For mixed fatal and non-fatal outcomes, ≤ 15 per 1000 will be considered trivial, 16–30 per 1000 will be considered small but important, and 31–45 per 1000 will be considered moderate in size. For continuous patient-reported quality of life measures, will search the literature for anchor-based minimal important difference (MID) estimates, and if no MID is identified we will use half the baseline standard deviation from normative data for the quality of life measure [45–47]. As per GRADE guidance, we will present our data in summary of findings tables [43].

We will conduct subgroup analyses or meta-regression, if appropriately powered, with a chi-square test of interaction to assess the following anticipated effect modifiers:

i) We will consider the primary macronutrient replacing the dietary fat under investigation (e.g., replacement of saturated fatty acid (SFA) with PUFA, MUFA, protein or carbohydrates). We anticipate that, for instance, replacing SFA with PUFA or MUFA will reduce the risk of cardiovascular events [32, 48] more than SFA reduction alone, or replacement with CHO or protein.
ii) We will conduct subgroups among RCTs that provide food (e.g., nuts, olive oil, fish) or fat supplementation versus those with dietary advice only, anticipating larger treatment effects in trials that provide food/supplementation interventions [32, 49].
iii) We will explore co-interventions as an effect modifier (e.g., statins, blood pressure lowering agents, exercise, or behavioral support groups), anticipating larger treatment effects in trials that provide active co-interventions [50, 51].
iv) We will use meta-regression to examine the association between the change in low density lipoprotein (LDL) and total cholesterol, surrogates for SFA reduction, and the log relative risk changes for each of our outcomes. We anticipate that participants with lower cholesterol levels will have a lower risk of stroke, myocardial infarction, coronary heart disease, and cardiovascular mortality.
v) Based on a modified version of the Cochrane risk of bias 1.0 instrument [28], we explore if studies at lower RoB have estimates of effect that differ significantly from studies at higher RoB, anticipating that studies at higher risk of bias will have larger treatment effects [52].

We will conduct a number of sensitivity analysis including (a) for primary studies that report on measures of dietary fats from participant-reported dietary intake surveys (i.e., dietary records or recalls, food frequency questionnaires) versus those that report tissue biomarkers (adipose polyunsaturated linoleic acid levels, subcutaneous fat aspirate, plasma fatty acid concentration) with or without participant-reported dietary intake assessments. While the number of established nutritional biomarkers is small, biomarkers may be applied directly in disease association analyses as has been done successfully for dairy fats [53] and alpha linolenic acid [54] based on valid markers for omaga 3 fatty acids [55], and may be used to calibrate self-report assessments to reduce systematic and random measurement error [56]. We will also conduct sensitivity analysis for (b) studies that report results corresponding to intake of dietary fats in absolute quantities (i.e., g/day) and those that report results from energy density models (i.e., % energy).

We will conduct pairwise meta-analyses using Revman 5.0, the meta package [57] and R version 3.5.1 (R Foundation for Statistical Computing).

### Evaluation of the certainty of evidence

We will assess the certainty of the evidence using the GRADE fully contextualized approach [24, 25] approach and present results for reducing and replacing/modifying saturated fat separately in a summary of findings table [43].

## Discussion

Our systematic review will provide a comprehensive and rigorous summary of the evidence addressing the relationship between dietary saturated and unsaturated fats and important health outcomes, including an estimate of the magnitude of effect, and the certainty of evidence.

This work represents a novel and efficient approach to evidence synthesis in fields in which many evidence syntheses have been previously published on dietary fats and health outcomes. Our systematic reviews will begin by utilizing the search strategies and selection of relevant studies done by existing well conducted systematic literature reviews (e.g., [32]), and subsequently we will search for primary studies from the date of the last systematic review forward.

### Implications

Our findings will be used towards the development of dietary guidelines addressing saturated fats and the risk of cardiovascular and cancer outcomes, including recommendations for those at very low, low, and high risk of a cardiovascular event.

### Dissemination

We will disseminate our findings by publication in a peer reviewed journal and by presentation at national and international conferences. Based on the findings of our overview, we will make GRADE summary of findings tables, plain language summaries, and infographics in user-friendly and open-access outputs for clinicians and patients and community members.

### Strengths and limitations

The strengths of this systematic review will include an extensive search for relevant systematic reviews of RCTs and RCTs; duplicate screening and extraction of data; the assessment of the RoB of studies using rigorous criteria aligned with advances in the methodology of bias assessment [28, 35, 37] including the assessment of the impact of missing participant outcome data on the RoB [27], and the transparent assessment of the magnitude and certainty of estimates for each of our outcomes using GRADE criteria [24, 25]. The GRADE approach to assessing the certainty of estimates is based on comprehensive methodology that has been described in detail in a series of eight *BMJ* publications and over 30 publications in the *Journal of Clinical Epidemiology* and has been adopted by over 110 international organizations, including the Cochrane Collaboration, Joanna Briggs Institute and the World Health Organization, each of which regularly apply GRADE to nutritional questions [11, 21]. The application of the GRADE approach will facilitate the consideration of important criteria that bear on the certainty of evidence on the relationship between saturated fats and health outcomes, including RoB, inconsistency, indirectness, imprecision, and publication bias. All this will serve to improve public transparency in making and communicating judgments about the magnitude and certainty of evidence on dietary fat and health outcomes.

## Conclusions

Our review will provide a comprehensive and rigorous summary of the evidence addressing the relationship between saturated, polyunsaturated and monounsaturated fats, and important health outcomes, including absolute estimates of the magnitude of effect and the certainty of evidence for these effects. Our findings will be used to establish dietary recommendations on saturated fat consumption, including reduction versus replacement of SFA with PUFA, MUFA, CHO, and protein.

## Data Availability

No additional data available.
